# Supplementary material for: A tropomyosin receptor kinase family protein, NTRK2 is a potential predictive biomarker for lung adenocarcinoma
Source: PeerJ. 2019 Jun 17;7:e7125. doi: 10.7717/peerj.7125 (PMC6585899; doi:10.7717/peerj.7125)
Supplement: Supplemental Information 1 — Figure S1. A Flow chart of analysis on the roles of NTRK2 in LUAD tumorigenesis. Figure S2. The negative association between the expression of NTRK2 and ERBB2 or MET in LUAD. Table S1: The main bioinformatics tools applied to analyze the role of NTRK2 in LUAD biological processes. Table S2: The methylation values of CpG islands in NTRK2. Table S3: The NTRK2-associated co-DEGs in LUAD. Table S4: The KEGG pathway of NTRK2-associated co-DEGs. [file peerj-07-7125-s001.docx]

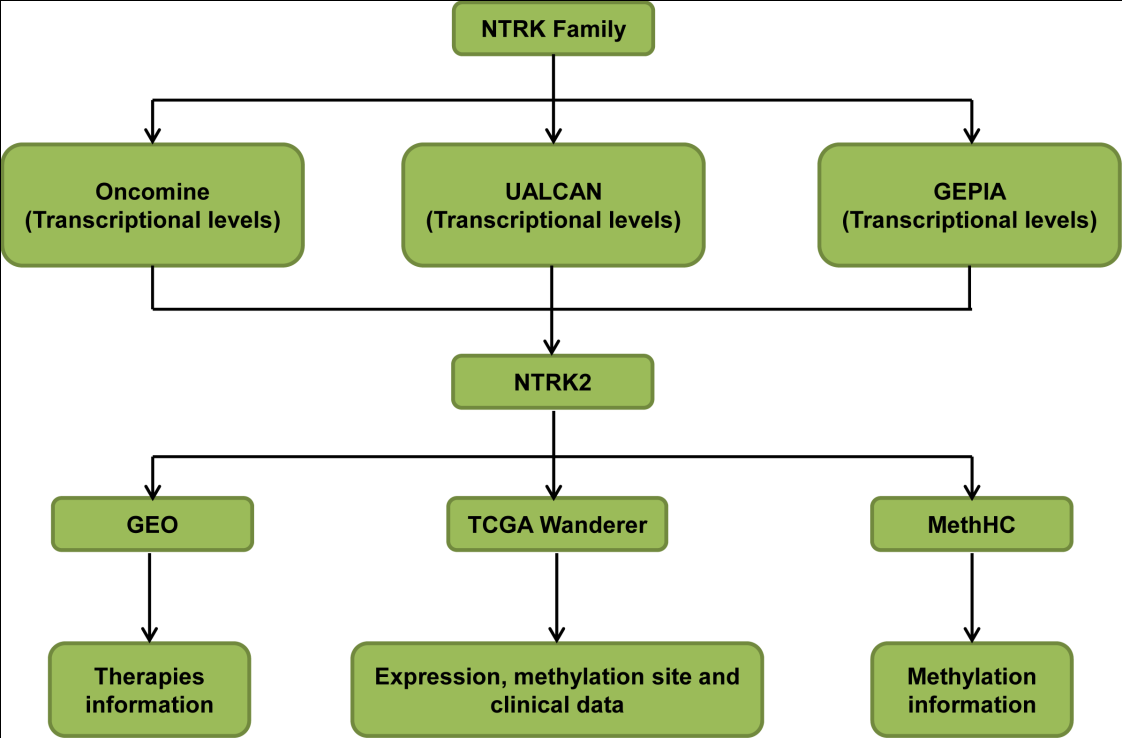


**Figure S1. A Flow chart of analysis on the roles of NTRK2 in LUAD tumorigenesis.** Using several public gene expression and methylation databases, we identified the low-expressed and high-methylated level of NTRK2 in LUAD patients.


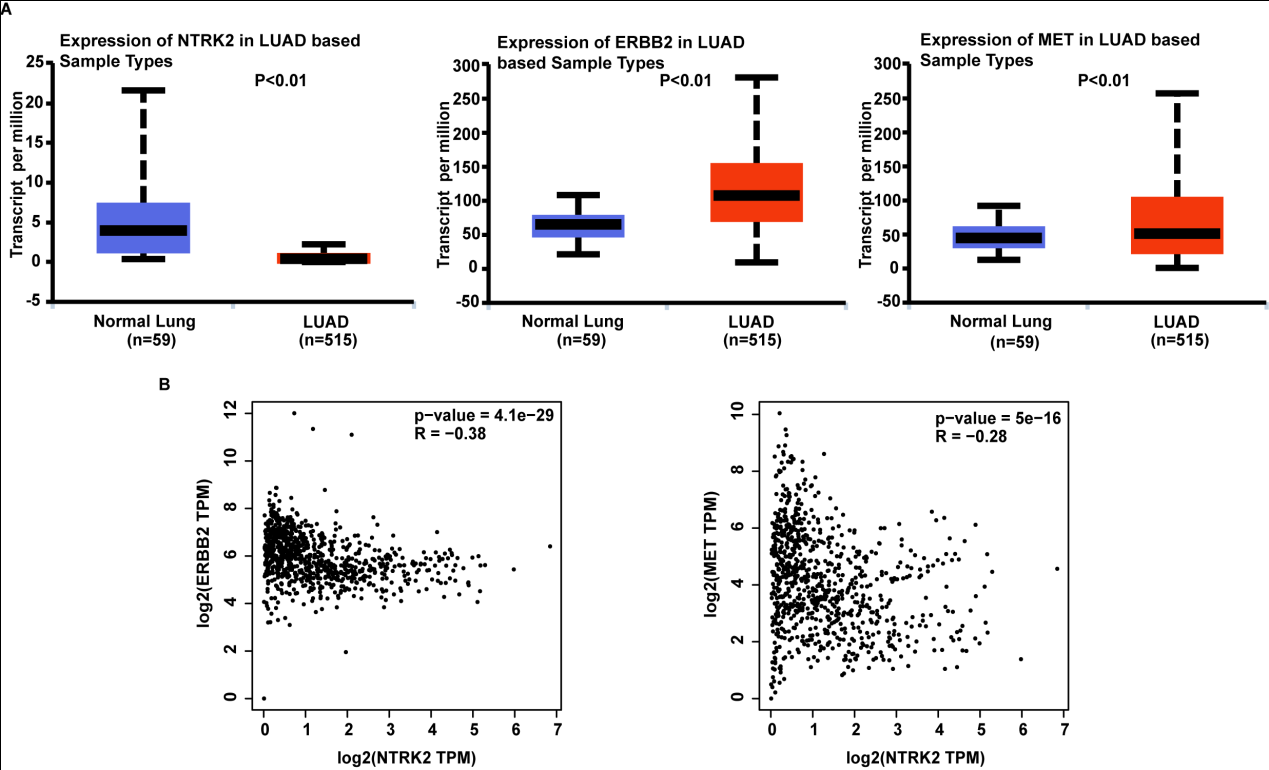


**Figure S2. The negative association between the expression of NTRK2 and ERBB2 or MET in LUAD.** (A) The mRNA expressions of NTRK2, ERBB2 and MET were evaluated from the database UALCAN. (B) The spearman correlation analysis between the expression of NTRK2 and ERBB2 or MET in LUAD.

Table S1: The main bioinformatics tools applied to analyze the role of NTRK2 in LUAD biological processes.

| **Databases** | **URL** | **Refs** |
| --- | --- | --- |
| Oncomine | https://www.oncomine.org/resource/login.html | [Rhodes et al. 2004] |
| GEO | https://www.ncbi.nlm.nih.gov/geoprofiles/ | [Barrett & Edgar 2008] |
| GEPIA | http://gepia.cancer-pku.cn/ | [Tang et al. 2017a] |
| GE-mini APP | http://gemini.cancer-pku.cn/ | [Tang et al. 2017b] |
| CRN | http://syslab4.nchu.edu.tw/index.jsp | [Li et al. 2016] |
| UALCAN | http://ualcan.path.uab.edu/index.html | [Chandrashekar et al. 2017] |
| Wanderer | http://maplab.imppc.org/wanderer/ | [Diez-Villanueva et al. 2015] |
| Kaplan-Meier plotter | http://kmplot.com/analysis/ | [Wang et al. 2018a] |
| MethHC | http://methhc.mbc.nctu.edu.tw/php/index.php | [Huang et al. 2015] |
| MethSurv | https://biit.cs.ut.ee/methsurv/ | [Modhukur et al. 2018] |
| cBioPortal | http://www.cbioportal.org/ | [Gao et al. 2013] |
| STRING | https://string-db.org/cgi/input.pl | [Szklarczyk et al. 2017] |
| Cytoscape | https://cytoscape.org/ | [Reimand et al. 2019] |
| DAVID | https://david.ncifcrf.gov/ | [Huang da et al. 2009] |
| WebGestalt | http://www.webgestalt.org/ | [Wang et al. 2017] |
| PATHVIEW | https://pathview.uncc.edu/analysis | [Luo et al. 2017] |
| KEGG  TCGA | <https://www.kegg.jp/>  https://cancergenome.nih.gov/ | [Kanehisa & Goto 2000]  [Hutter & Zenklusen 2018] |

Table S2: The methylation values of CpG islands in NTRK2.

| **probe** | **chr** | **cg-start** | **cg-end** | **Tum-mean** | **Tum sd** | **Wilcox-stat** | **P-value** |
| --- | --- | --- | --- | --- | --- | --- | --- |
| **cg03628748** | **chr9** | **87285133** | **87285134** | **0.302257966** | **0.135415025** | **1988** | **4.35E-12** |
| cg08470639 | chr9 | 87285186 | 87285187 | 0.232001928 | 0.116967579 | 2157.5 | 1.96E-11 |
| cg09926027 | chr9 | 87285693 | 87285694 | 0.280837705 | 0.122622151 | 2753 | 2.72E-09 |
| cg13723118 | chr9 | 87284722 | 87284723 | 0.208108029 | 0.138424277 | 2977 | 1.50E-08 |
| cg01009697 | chr9 | 87283470 | 87283471 | 0.12862715 | 0.16147128 | 3855 | 5.64E-06 |
| cg13965062 | chr9 | 87284706 | 87284707 | 0.260734715 | 0.081993129 | 3836 | 5.02E-06 |
| cg09539438 | chr9 | 87283789 | 87283790 | 0.13419738 | 0.109689173 | 4742 | 0.000658875 |
| cg01292475 | chr9 | 87284571 | 87284572 | 0.137075676 | 0.095355043 | 5153 | 0.003964883 |
| cg13620631 | chr9 | 87489528 | 87489529 | 0.381875592 | 0.13938745 | 8961 | 0.047268959 |
| cg14447193 | chr9 | 87433864 | 87433865 | 0.697653704 | 0.129733117 | 6060 | 0.085083937 |
| cg13504245 | chr9 | 87282610 | 87282611 | 0.15836126 | 0.150723689 | 6589 | 0.295592948 |
| cg13654445 | chr9 | 87636383 | 87636384 | 0.839578577 | 0.124580195 | 6564 | 0.281088449 |
| cg22402007 | chr9 | 87282823 | 87282824 | 0.267598702 | 0.15008895 | 6656 | 0.336897179 |
| cg13698224 | chr9 | 87309394 | 87309395 | 0.522148187 | 0.191332872 | 8105 | 0.373448924 |

Table S3: The NTRK2-associated co-DEGs in LUAD.

| **Gene** | **Cytoband** | **Log Ratio** | **P-Value** | **Q-Value** | **Tendency** |
| --- | --- | --- | --- | --- | --- |
| MFSD3 | 8q24.3 | -2.04 | 2.12E-11 | 1.31E-07 | Under-expressed |
| OVGP1 | 1p13.2 | 3.11 | 6.48E-11 | 2.46E-07 | Over-expressed |
| C9ORF116 | 9q34.3 | -2.11 | 2.94E-10 | 8.14E-07 | Under-expressed |
| ADGRG1 | 16q21 | -2.6 | 3.23E-10 | 8.14E-07 | Under-expressed |
| PTGER2 | 14q22.1 | 2.64 | 1.09E-09 | 1.83E-06 | Over-expressed |
| PRR7 | 5q35.3 | -2.41 | 1.88E-09 | 2.59E-06 | Under-expressed |
| SLC34A3 | 9q34.3 | -2.48 | 2.16E-09 | 2.73E-06 | Under-expressed |
| SHISA3 | 4p13 | 6.15 | 2.77E-09 | 3.23E-06 | Over-expressed |
| ZNF614 | 19q13.41 | 2.01 | 4.21E-09 | 4.21E-06 | Over-expressed |
| DOK4 | 16q21 | 2.9 | 6.03E-09 | 4.62E-06 | Over-expressed |
| HNF1B | 17q12 | 2.04 | 6.03E-09 | 4.62E-06 | Over-expressed |
| NPAS1 | 19q13.32 | -2.18 | 9.59E-09 | 6.52E-06 | Under-expressed |
| CDH26 | 20q13.33 | -4.22 | 3.14E-08 | 1.80E-05 | Under-expressed |
| CLDN10 | 13q32.1 | -5.23 | 4.42E-08 | 2.16E-05 | Under-expressed |
| WNK4 | 17q21.2 | 3.2 | 4.43E-08 | 2.16E-05 | Over-expressed |
| TMPRSS6 | 22q12.3 | -4.91 | 4.56E-08 | 2.16E-05 | Under-expressed |
| SCUBE1 | 22q13.2 | 3.3 | 8.36E-08 | 3.17E-05 | Over-expressed |
| ALDH3B2 | 11q13.2 | -3.45 | 8.59E-08 | 3.17E-05 | Under-expressed |
| TOX | 8q12.1 | 2.35 | 9.11E-08 | 3.21E-05 | Over-expressed |
| FADS2 | 11q12.2 | -2.35 | 1.16E-07 | 3.79E-05 | Under-expressed |
| HTR3A | 11q23.2 | -5.08 | 1.44E-07 | 4.35E-05 | Under-expressed |
| ARHGAP20 | 11q22.3-q23.1 | 2.53 | 2.59E-07 | 6.77E-05 | Over-expressed |
| S100A1 | 1q21.3 | 2.02 | 2.86E-07 | 7.24E-05 | Over-expressed |
| MUC5B | 11p15.5 | -4.88 | 4.57E-07 | 1.00E-04 | Under-expressed |
| NCAPG | 4p15.31 | -2.02 | 4.93E-07 | 1.04E-04 | Under-expressed |
| KLKB1 | 4q35.2 | 2.24 | 5.50E-07 | 1.14E-04 | Over-expressed |
| GGTLC1 | 20p11.21 | 3.88 | 5.64E-07 | 1.15E-04 | Over-expressed |
| HIST1H3D | 6p22.2 | -3.1 | 6.77E-07 | 1.32E-04 | Under-expressed |
| CCDC78 | 16p13.3 | -2.52 | 7.37E-07 | 1.38E-04 | Under-expressed |
| TMEM233 | 12q24.23 | 3.15 | 8.73E-07 | 1.50E-04 | Over-expressed |
| SLC1A7 | 1p32.3 | 5.14 | 1.13E-06 | 1.76E-04 | Over-expressed |
| CHL1 | 3p26.3 | -3.19 | 1.23E-06 | 1.88E-04 | Under-expressed |
| MUC15 | 11p14.2 | 3.68 | 1.29E-06 | 1.95E-04 | Over-expressed |
| SLC44A5 | 1p31.1 | -4.33 | 1.42E-06 | 2.08E-04 | Under-expressed |
| RSPH9 | 6p21.1 | -2.16 | 1.65E-06 | 2.29E-04 | Under-expressed |
| SCN8A | 12q13.13 | -2.61 | 1.83E-06 | 2.47E-04 | Under-expressed |
| MLXIPL | 7q11.23 | -2.92 | 2.11E-06 | 2.76E-04 | Under-expressed |
| PIMREG | 17p13.2 | -2.12 | 2.36E-06 | 3.00E-04 | Under-expressed |
| PROM2 | 2q11.1 | -2.32 | 5.26E-06 | 5.61E-04 | Under-expressed |
| FAM149A | 4q35.1 | 2.11 | 5.56E-06 | 5.85E-04 | Over-expressed |
| KISS1R | 19p13.3 | -3.06 | 6.31E-06 | 6.42E-04 | Under-expressed |
| UGT8 | 4q26 | 2.75 | 6.42E-06 | 6.42E-04 | Over-expressed |
| C3ORF67 | 3p14.2 | -2.13 | 7.50E-06 | 7.24E-04 | Under-expressed |
| COL4A3 | 2q36.3 | 3.07 | 7.92E-06 | 7.54E-04 | Over-expressed |
| SNCG | 10q23.2 | -2.47 | 8.89E-06 | 8.03E-04 | Under-expressed |
| ROBO2 | 3p12.3 | 2.7 | 9.16E-06 | 8.16E-04 | Over-expressed |
| SLCO4A1 | 20q13.33 | -2.15 | 9.59E-06 | 8.36E-04 | Under-expressed |
| CCDC85A | 2p16.1 | 2 | 1.02E-05 | 8.68E-04 | Over-expressed |
| CIT | 12q24.23 | -2.79 | 1.13E-05 | 9.30E-04 | Under-expressed |
| CSPG5 | 3p21.31 | 3.83 | 1.36E-05 | 1.04E-03 | Over-expressed |
| LIMS3-LOC440895 | 2q13 | -2.16 | 1.51E-05 | 1.11E-03 | Under-expressed |
| ELN | 7q11.23 | 2.3 | 1.60E-05 | 1.16E-03 | Over-expressed |
| AKR1B10 | 7q33 | -4.54 | 1.63E-05 | 1.17E-03 | Under-expressed |
| KRT16 | 17q21.2 | -3.66 | 1.68E-05 | 1.20E-03 | Under-expressed |
| KIF12 | 9q32 | 2.54 | 1.74E-05 | 1.22E-03 | Over-expressed |
| SDK1 | 7p22.2 | -2.08 | 1.86E-05 | 1.29E-03 | Under-expressed |
| TLR3 | 4q35.1 | 2.05 | 1.96E-05 | 1.33E-03 | Over-expressed |
| SEMA3E | 7q21.11 | -3.09 | 2.06E-05 | 1.38E-03 | Under-expressed |
| ZNF114 | 19q13.33 | 3.17 | 2.35E-05 | 1.52E-03 | Over-expressed |
| CARD14 | 17q25.3 | -2.45 | 2.56E-05 | 1.60E-03 | Under-expressed |
| CDKL2 | 4q21.1 | 3.63 | 2.69E-05 | 1.64E-03 | Over-expressed |
| TMEM59L | 19p13.11 | -3.56 | 2.87E-05 | 1.71E-03 | Under-expressed |
| SCG5 | 15q13.3 | -2.52 | 3.08E-05 | 1.81E-03 | Under-expressed |
| CNGA4 | 11p15.4 | -2.32 | 3.19E-05 | 1.86E-03 | Under-expressed |
| CCDC151 | 19p13.2 | -2.32 | 3.61E-05 | 2.02E-03 | Under-expressed |
| LAIR2 | 19q13.42 | 2 | 3.94E-05 | 2.17E-03 | Over-expressed |
| DEGS2 | 14q32.2 | -3.14 | 4.00E-05 | 2.17E-03 | Under-expressed |
| THBS4 | 5q14.1 | 2.48 | 5.02E-05 | 2.62E-03 | Over-expressed |
| TFF3 | 21q22.3 | -4.9 | 5.13E-05 | 2.65E-03 | Under-expressed |
| SYTL5 | Xp11.4 | -2.72 | 5.75E-05 | 2.90E-03 | Under-expressed |
| GFRA3 | 5q31.2 | 3.6 | 6.34E-05 | 3.15E-03 | Over-expressed |
| LOC388242 | 16p11.2 | -2.28 | 7.60E-05 | 3.61E-03 | Under-expressed |
| LGALS12 | 11q12.3 | -2.37 | 7.81E-05 | 3.64E-03 | Under-expressed |
| PLLP | 16q13 | 2.09 | 8.22E-05 | 3.79E-03 | Over-expressed |
| FAM189A2 | 9q21.12 | 2.05 | 8.59E-05 | 3.90E-03 | Over-expressed |
| SLAMF9 | 1q23.2 | -2.2 | 8.60E-05 | 3.90E-03 | Under-expressed |
| ATP7B | 13q14.3 | -2.15 | 1.02E-04 | 4.42E-03 | Under-expressed |
| CES4A | 16q22.1 | -2.32 | 1.06E-04 | 4.56E-03 | Under-expressed |
| DYNLRB2 | 16q23.2 | -2.07 | 1.12E-04 | 4.72E-03 | Under-expressed |
| SAA1 | 11p15.1 | -2.42 | 1.16E-04 | 4.81E-03 | Under-expressed |
| CHRNA5 | 15q25.1 | -2.7 | 1.27E-04 | 5.21E-03 | Under-expressed |
| RCAN2 | 6p12.3 | 3.99 | 2.02E-04 | 7.14E-03 | Over-expressed |
| PCP4L1 | 1q23.3 | 2.28 | 2.02E-04 | 7.14E-03 | Over-expressed |
| UMODL1 | 21q22.3 | -3.97 | 2.56E-04 | 8.42E-03 | Under-expressed |
| ZNF682 | 19p12 | 2.07 | 2.94E-04 | 9.32E-03 | Over-expressed |
| ADGRF1 | 6p12.3\|6 | 2.93 | 2.95E-04 | 9.32E-03 | Over-expressed |
| GJB1 | Xq13.1 | 3.73 | 3.58E-04 | 0.0108 | Over-expressed |
| RNASE1 | 14q11.2 | 2.59 | 4.01E-04 | 0.0117 | Over-expressed |
| FXYD1 | 19q13.12 | 2.41 | 4.26E-04 | 0.0123 | Over-expressed |
| MDH1B | 2q33.3 | -2.39 | 4.28E-04 | 0.0123 | Under-expressed |
| KCNH8 | 3p24.3 | -2.31 | 4.32E-04 | 0.0124 | Under-expressed |
| TRIM6 | 11p15.4 | 2.99 | 4.44E-04 | 0.0127 | Over-expressed |
| PIGR | 1q32.1 | 2.65 | 4.99E-04 | 0.0139 | Over-expressed |
| HLF | 17q22 | 3.59 | 5.21E-04 | 0.0143 | Over-expressed |
| GRIN3B | 19p13.3 | -2.98 | 5.57E-04 | 0.0151 | Under-expressed |
| GCLC | 6p12.1 | -2.59 | 5.65E-04 | 0.0152 | Under-expressed |
| PCDHB2 | 5q31.3 | -2.11 | 5.91E-04 | 0.0157 | Under-expressed |
| GATA5 | 20q13.33 | 2 | 5.97E-04 | 0.0159 | Over-expressed |
| IRX2 | 5p15.33 | 2.64 | 6.34E-04 | 0.0167 | Over-expressed |
| ANO5 | 11p14.3 | 2.81 | 6.39E-04 | 0.0168 | Over-expressed |
| PALM3 | 19p13.12 | 2.31 | 6.71E-04 | 0.0174 | Over-expressed |
| POU5F1 | 6p21.33 | 2.28 | 6.74E-04 | 0.0174 | Over-expressed |
| NCMAP | 1p36.11 | 2.82 | 6.79E-04 | 0.0175 | Over-expressed |
| B3GALT2 | 1q31.2 | 2.58 | 8.10E-04 | 0.02 | Over-expressed |
| SLC47A1 | 17p11.2 | 2.04 | 8.28E-04 | 0.0203 | Over-expressed |
| MAMDC2 | 9q21.12 | 2.22 | 9.86E-04 | 0.0232 | Over-expressed |
| RPS28 | 19p13.2 | 3.14 | 1.22E-03 | 0.027 | Over-expressed |
| FREM2 | 13q13.3 | 2.14 | 1.23E-03 | 0.0272 | Over-expressed |
| LRRC46 | 17q21.32 | -2.32 | 1.36E-03 | 0.0293 | Under-expressed |
| MISP | 19p13.3 | -3.96 | 1.36E-03 | 0.0293 | Under-expressed |
| TEKT2 | 1p34.3 | -2.23 | 1.36E-03 | 0.0293 | Under-expressed |
| PLD4 | 14q32.33 | 2.04 | 1.37E-03 | 0.0294 | Over-expressed |
| LRRC15 | 3q29 | 2.11 | 1.38E-03 | 0.0294 | Over-expressed |
| TF | 3q22.1 | -3.7 | 1.43E-03 | 0.0304 | Under-expressed |
| KBTBD12 | 3q21.3 | 2.27 | 1.43E-03 | 0.0304 | Over-expressed |
| ADRB1 | 10q25.3 | 2.89 | 1.51E-03 | 0.0314 | Over-expressed |
| PHACTR1 | 6p24.1 | 2.88 | 1.62E-03 | 0.0331 | Over-expressed |
| C1QTNF7 | 4p15.32 | 2.25 | 1.65E-03 | 0.0333 | Over-expressed |
| CHIA | 1p13.2 | 5.61 | 1.72E-03 | 0.0344 | Over-expressed |
| FRMD4B | 3p14.1 | 2.03 | 1.79E-03 | 0.0354 | Over-expressed |
| CEACAM7 | 19q13.2 | -2.38 | 1.86E-03 | 0.0365 | Under-expressed |
| CYP24A1 | 20q13.2 | -2.36 | 1.96E-03 | 0.038 | Under-expressed |
| TMPRSS4 | 11q23.3 | -5.98 | 2.06E-03 | 0.0394 | Under-expressed |
| MUC16 | 19p13.2 | -5.26 | 2.15E-03 | 0.0407 | Under-expressed |
| HP | 16q22.2 | -2.65 | 2.19E-03 | 0.041 | Under-expressed |
| HYAL1 | 3p21.31 | 2.96 | 2.21E-03 | 0.0413 | Over-expressed |
| VAX2 | 2p13.3 | -2.01 | 2.28E-03 | 0.0422 | Under-expressed |
| DCDC2 | 6p22.3 | 2.05 | 2.33E-03 | 0.0428 | Over-expressed |
| CLIC6 | 21q22.12 | -2.24 | 2.41E-03 | 0.0438 | Under-expressed |
| PTPN13 | 4q21.3 | 2.6 | 2.49E-03 | 0.0449 | Over-expressed |
| GSTA1 | 6p12.2 | -4.05 | 2.71E-03 | 0.0474 | Under-expressed |
| KLK10 | 19q13.41 | -2.72 | 3.28E-03 | 0.0544 | Under-expressed |
| SULT2B1 | 19q13.33 | -3.23 | 3.47E-03 | 0.0565 | Under-expressed |
| SDR16C5 | 8q12.1 | 2.13 | 3.57E-03 | 0.0575 | Over-expressed |
| POM121L10P | 22q11.23 | 2.32 | 3.63E-03 | 0.0581 | Over-expressed |
| PPP2R2C | 4p16.1 | -2.76 | 3.67E-03 | 0.0586 | Under-expressed |
| PCSK9 | 1p32.3 | -2.4 | 3.80E-03 | 0.06 | Under-expressed |
| TRNP1 | 1p36.11 | -2.57 | 3.91E-03 | 0.0608 | Under-expressed |
| AKR7A3 | 1p36.13 | -2.09 | 4.12E-03 | 0.0631 | Under-expressed |
| C6ORF141 | 6p12.3 | -2.24 | 4.64E-03 | 0.0695 | Under-expressed |
| COL9A2 | 1p34.2 | 2.04 | 5.06E-03 | 0.0739 | Over-expressed |
| YBX2 | 17p13.1 | -4.1 | 5.14E-03 | 0.0747 | Under-expressed |
| CXCL6 | 4q13.3 | -2.87 | 5.25E-03 | 0.0761 | Under-expressed |
| TCN1 | 11q12.1 | -2.32 | 5.80E-03 | 0.0823 | Under-expressed |
| DTX1 | 12q24.13 | -2.09 | 5.90E-03 | 0.0832 | Under-expressed |
| UGT1A6 | 2q37.1 | -2.98 | 6.23E-03 | 0.0867 | Under-expressed |
| NAPSA | 19q13.33 | 2.05 | 6.33E-03 | 0.0877 | Over-expressed |
| PPP1R3C | 10q23.32 | 2.5 | 6.41E-03 | 0.0883 | Over-expressed |
| DNAJB13 | 11q13.4 | 2.19 | 7.39E-03 | 0.0989 | Over-expressed |
| TNS4 | 17q21.2 | -2.05 | 7.49E-03 | 0.1 | Under-expressed |
| TUBB3 | 16q24.3 | -2.95 | 7.51E-03 | 0.1 | Under-expressed |
| CCDC74B | 2q21.1 | -2.5 | 7.86E-03 | 0.104 | Under-expressed |
| DNAH11 | 7p15.3 | 2.62 | 8.12E-03 | 0.106 | Over-expressed |
| INHBB | 2q14.2 | -3 | 8.40E-03 | 0.108 | Under-expressed |
| MGLL | 3q21.3 | 2.1 | 8.42E-03 | 0.108 | Over-expressed |
| PON3 | 7q21.3 | 2.19 | 8.72E-03 | 0.111 | Over-expressed |
| C1QTNF12 | 1p36.33 | -2.05 | 8.91E-03 | 0.113 | Under-expressed |
| STEAP2 | 7q21.13 | -2.73 | 9.02E-03 | 0.114 | Under-expressed |
| AQP4 | 18q11.2 | 3.23 | 9.60E-03 | 0.119 | Over-expressed |
| DRAIC | 15q23 | -3.16 | 0.0102 | 0.125 | Under-expressed |
| AFAP1-AS1 | 4p16.1 | 2.3 | 0.0107 | 0.129 | Over-expressed |
| PRRX2 | 9q34.11 | 2.35 | 0.0112 | 0.134 | Over-expressed |
| SNAP25 | 20p12.2 | -2.14 | 0.0113 | 0.134 | Under-expressed |
| RNF217-AS1 | 6q22.31 | 2.31 | 0.0114 | 0.135 | Over-expressed |
| FOLR1 | 11q13.4 | 2.22 | 0.0116 | 0.136 | Over-expressed |
| ARHGAP31 | 3q13.32-q13.33 | 2.15 | 0.0118 | 0.138 | Over-expressed |
| RAP1GAP | 1p36.12 | 2.44 | 0.0121 | 0.14 | Over-expressed |
| ERN2 | 16p12.2 | -5.05 | 0.0133 | 0.15 | Under-expressed |
| CYP2D6 | 22q13.2 | 3.03 | 0.0154 | 0.166 | Over-expressed |
| B3GNT6 | 11q13.5 | -6.99 | 0.0156 | 0.167 | Under-expressed |
| ARX | Xp21.3 | 4.75 | 0.0159 | 0.169 | Over-expressed |
| HOXD1 | 2q31.1 | 4.64 | 0.0163 | 0.172 | Over-expressed |
| CFAP45 | 1q23.2 | -2.98 | 0.0165 | 0.173 | Under-expressed |
| IP6K3 | 6p21.31 | -3.07 | 0.0176 | 0.181 | Under-expressed |
| MS4A2 | 11q12.1 | 2.24 | 0.0177 | 0.181 | Over-expressed |
| CX3CR1 | 3p22.2 | 2.03 | 0.0177 | 0.182 | Over-expressed |
| KCNG1 | 20q13.13 | -2.07 | 0.0179 | 0.182 | Under-expressed |
| GGH | 8q12.3 | -2.35 | 0.0179 | 0.182 | Under-expressed |
| UBE2C | 20q13.12 | -2.53 | 0.0181 | 0.183 | Under-expressed |
| CFAP206 | 6q15 | -2.01 | 0.0188 | 0.189 | Under-expressed |
| AUTS2 | 7q11.22 | 2.31 | 0.021 | 0.204 | Over-expressed |
| CLEC4F | 2p13.3 | 2.14 | 0.0213 | 0.206 | Over-expressed |
| ZNF486 | 19p12 | 2.14 | 0.0214 | 0.206 | Over-expressed |
| ATP6V1B1 | 2p13.3 | -2.48 | 0.0221 | 0.211 | Under-expressed |
| SPTSSB | 3q26.1 | -2.63 | 0.0225 | 0.215 | Under-expressed |
| C2CD6 | 2q33.1 | 2.74 | 0.023 | 0.217 | Over-expressed |
| HGD | 3q13.33 | -5.33 | 0.0232 | 0.22 | Under-expressed |
| GGN | 19q13.2 | -2.16 | 0.0242 | 0.225 | Under-expressed |
| GPR37 | 7q31.33 | -2.51 | 0.0269 | 0.244 | Under-expressed |
| PSCA | 8q24.3 | -4.85 | 0.0298 | 0.263 | Under-expressed |
| PLPPR1 | 9q31.1 | 2.7 | 0.0314 | 0.271 | Over-expressed |
| TRIM17 | 1q42.13 | -2.81 | 0.0316 | 0.272 | Under-expressed |
| CCDC153 | 11q23.3 | -2.52 | 0.0338 | 0.285 | Under-expressed |
| S100P | 4p16.1 | -6.62 | 0.0339 | 0.285 | Under-expressed |
| LOC153684 | 5p12 | 2.16 | 0.0356 | 0.294 | Over-expressed |
| ADH1B | 4q23 | 2.12 | 0.0357 | 0.294 | Over-expressed |
| SIGLEC8 | 19q13.41 | 2.7 | 0.0365 | 0.299 | Over-expressed |
| APOBEC2 | 6p21.1 | 6.03 | 0.0374 | 0.303 | Over-expressed |
| SLC16A11 | 17p13.1 | -2.3 | 0.0379 | 0.305 | Under-expressed |
| EFR3B | 2p23.3 | 2.17 | 0.0388 | 0.31 | Over-expressed |
| LONRF2 | 2q11.2 | -2.54 | 0.0388 | 0.31 | Under-expressed |
| CCL7 | 17q12 | -2.39 | 0.0389 | 0.31 | Under-expressed |
| MST1L | 1p36.13 | 3.75 | 0.0389 | 0.31 | Over-expressed |
| FSCN2 | 17q25.3 | -2.74 | 0.0397 | 0.314 | Under-expressed |
| PLEKHG5 | 1p36.31 | -2 | 0.0406 | 0.319 | Under-expressed |
| DLGAP1 | 18p11.31 | 2.42 | 0.0434 | 0.335 | Over-expressed |
| SLC4A3 | 2q35 | -2.87 | 0.0437 | 0.336 | Under-expressed |
| UPB1 | 22q11.23 | 2.34 | 0.0439 | 0.337 | Over-expressed |
| VSIG1 | Xq22.3 | -4.93 | 0.0442 | 0.338 | Under-expressed |
| CX3CL1 | 16q21 | 2.56 | 0.0442 | 0.338 | Over-expressed |
| DEPDC1B | 5q12.1 | -2.27 | 0.0446 | 0.34 | Under-expressed |
| C4BPA | 1q32.2 | 3.11 | 0.0447 | 0.34 | Over-expressed |
| SCTR | 2q14.2 | 4.05 | 0.0453 | 0.343 | Over-expressed |
| TSPAN1 | 1p34.1 | -4.13 | 0.0455 | 0.344 | Under-expressed |
| PTGFR | 1p31.1 | -2.73 | 0.0484 | 0.36 | Under-expressed |
| VNN3 | 6q23.2 | -2.82 | 0.0488 | 0.362 | Under-expressed |
| LYPD6B | 2q23.2 | -4.84 | 0.0496 | 0.366 | Under-expressed |
| RNF128 | Xq22.3 | 2.47 | 0.0497 | 0.367 | Over-expressed |

Table S4: The KEGG pathway of NTRK2-associated co-DEGs.

| **Term** | **Count** | **PValue** | **Genes** |
| --- | --- | --- | --- |
| hsa00980:Metabolism of xenobiotics by cytochrome P450 | 6 | 0.00136 | GSTA1, UGT1A6, CYP2D6, AKR7A3, ADH1B, ALDH3B2 |
| hsa00982:Drug metabolism - cytochrome P450 | 5 | 0.006879 | GSTA1, UGT1A6, CYP2D6, ADH1B, ALDH3B2 |
